# Complete Genome Sequence of *Cupriavidus basilensis* 4G11, Isolated from the Oak Ridge Field Research Center Site

**DOI:** 10.1128/genomeA.00322-15

**Published:** 2015-05-14

**Authors:** Jayashree Ray, R. Jordan Waters, Jeffrey M. Skerker, Jennifer V. Kuehl, Morgan N. Price, Jiawen Huang, Romy Chakraborty, Adam P. Arkin, Adam Deutschbauer

**Affiliations:** a Physical Biosciences Division, Lawrence Berkeley National Laboratory, Berkeley, California, USA; b Joint Genome Institute, Lawrence Berkeley National Laboratory, Berkeley, California, USA; c Energy Biosciences Institute, University of California, Berkeley, Berkeley, California, USA; d Earth Sciences Division, Lawrence Berkeley National Laboratory, Berkeley, California, USA

## Abstract

*Cupriavidus basilensis* 4G11 was isolated from groundwater at the Oak Ridge Field Research Center (FRC) site. Here, we report the complete genome sequence and annotation of *Cupriavidus basilensis* 4G11. The genome contains 8,421,483 bp, 7,661 predicted protein-coding genes, and a total GC content of 64.4%.

## GENOME ANNOUNCEMENT

*Cupriavidus* species (previously known as *Wautersia* or *Ralstonia*) are Gram-negative betaproteobacteria that are known for their diverse metabolic capabilities (1–6). In addition, heavy-metal resistance is a typical characteristic of *Cupriavidus* strains; previously sequenced *Cupriavidus* species such as *C. metallidurans*, *C. eutrophus*, *C. pinatubonensis*, *C. taiwanensis*, and *Cupriavidus* sp. strain BIS7 encode a number of putative proteins involved in heavy-metal resistance and biodegradation activities (1–3, 5, 7–14). *Cupriavidus basilensis* 4G11 was isolated on R2A media from a single port well (area 5, FW 507) of the FRC at Oak Ridge, TN. *Cupriavidus basilensis* 4G11 has a 16S rRNA gene 99.1% identical to that of *Cupriavidus basilensis* strain DSM 11853.

Whole-genome sequencing of *Cupriavidus basilensis* 4G11 was done primarily with Pacific Biosciences (PacBio) SMRT sequencing. We generated 4 SMRT cells of sequencing data with an average insert size of 8 kb. For comparison and some error correction of the final PacBio assembly, we also collected Illumina sequencing data. For Illumina sequencing, a TruSeq DNA sample prep v2 kit (Illumina, Inc., CA) was used to prepare an Illumina paired-end library from an average insert size of 800 bp. A total of 456,136 paired-end reads (2 × 300 bp) were generated on an Illumina MiSeq instrument. Illumina sequence assembly was performed using CLC Genomics Workbench (version 7.5.1). We assembled the PacBio data with SMRT Portal software (SMRTAnalysis version v2.2.0.p2) and an HGAP2 assembly algorithm to an overall coverage estimate of 150×. We identified ~10 kbp of repeated sequence at the end of each PacBio scaffold that indicated the scaffold was a circular chromosome. We mapped the Illumina data to the PacBio scaffolds and then used the Illumina consensus sequence to replace the repeated PacBio regions on each scaffold. This resulted in two closed circular chromosomes. The assembled *Cupriavidus basilensis* 4G11 genome is 8,421,483 bp with two chromosomes of lengths of 4,522,716 bp (main) and 3,898,767 bp (secondary), and the total GC content is 64.4%.

We used the Rapid Annotation using Subsystem Technology (RAST) server (15) for annotating the *Cupriavidus basilensis* 4G11 genome. The entire genome contains 7,661 predicted protein-coding genes, 69 tRNAs (56 on the main chromosome, 13 on secondary chromosomes), and 21 rRNAs (including 23S, 16S, and 5S). The genome annotation reveals a number of putative proteins responsible for heavy-metal resistance, including CzcABD (putative cobalt, zinc, and cadmium resistance), CtpF (metal cation transporter), a lead-, cadmium-, mercury-, and zinc-transporting ATPase, and a copper-translocating P-type ATPase. The presence of these putative metal resistance proteins and the broad metabolic diversity of *Cupriavidus* make this organism relevant for biogeochemical and bioremediation studies.

### Nucleotide sequence accession numbers.

This whole-genome shotgun project has been deposited in GenBank under the accession numbers CP010536 (main chromosome) and CP010537 (secondary chromosome).
